# A Geodemographic View of the Accessibility of Selected Outpatient Services in Czechia

**DOI:** 10.3389/ijph.2022.1604067

**Published:** 2022-02-18

**Authors:** Kateřina Maláková

**Affiliations:** Department of Demography and Geodemography, Faculty of Science, Charles University, Prague, Czechia

**Keywords:** health services, accessibility, utilization, diabetology, cardiology, psychiatry, Czechia

## Abstract

**Objectives:** Although people use health services throughout their lives, there are important differences in timing, location, and frequency of utilization. The aim of this article is to identify and explain these differences in terms of healthcare accessibility.

**Methods:** Outpatient health services—diabetology, cardiology, and psychiatry—are analysed using anonymized data from the General Health Insurance Company (GHIC) in Czechia for 2019. Healthcare utilization is studied in relation to selected geodemographic characteristics—patient’s age, sex, place of permanent residence, and location of healthcare provision.

**Results:** The analysis found significant differences in the utilization of the selected health services in terms of age, sex, and size of the patient’s municipality of residence. Generally, men tended to travel outside their municipality for healthcare more than women. Young patients were more likely (and also further) to travel outside their municipality for healthcare than older patients.

**Conclusion:** The reasons for this were the location of the health service provider (mostly concentrated in local/regional centres), the patient’s ability and willingness to travel for healthcare, and differences in the patient’s permanent and ordinary place of residence.

## Introduction

Access to healthcare is one of the basic goals of healthcare systems around the world. Healthcare utilization, as access to health services, is determined by a number of individual and socioeconomic factors and the health system itself, not just the available and demand for them. The main factors limiting service use include cost, material and geographic accessibility, individual, social and cultural barriers, and the quality of the health services [1–3].

The focus of this article is on the geographical accessibility of health services, which is mainly affected by the spatial distribution of both the population using the service and the health service providers (HSP). Limited healthcare services in one geographic area can be compensated for by travelling to another [4, 5]. Rural areas typically have a limited choice of HSPs, and patients are forced to travel further for healthcare [6, 7].

The use of health services is affected by many factors, the most important demographic ones are sex and age [8, 9]. As individuals can use healthcare services throughout their life, it is generally the case that healthcare utilization increases as the individual grows older [10, 11]. Simultaneously, the share of older inhabitants, i.e., inhabitants with a higher probability of using health services, has increased over a long period that is closely correlative of population ageing. The population in Czechia, like other EU28 member states, has considerably aged. Current projections show that this trend will continue in the coming decades and could impact the healthcare utilization [12, 13].

Regarding sex differences, women tend to make more use of healthcare than men [14, 15]. The results of a study by Bertakis et al. [16] show that on average women visited primary healthcare clinics and diagnostic services much more than men. On the contrary, younger people and men are assumed to tend to travel further, including for health services. The distance to HSP plays an important role in healthcare utilization, and public transport could contribute to improving individual health and reducing health inequalities [17].

The aim of this article is to explore the basic geodemographic differences in the use of selected outpatient services in Czechia in 2019. Access to health services is analysed as the mean distance between the patient’s place of residence and the location of health service provided. The utilization and accessibility of health services is studied in relation to age, sex, patient’s place of permanent residence, and the location of HSP. The selected outpatient health services are diabetology, cardiology, and psychiatry, which all have a large number of patients and have seen continual growth in patients in recent years.

## Study Area

Czechia has a universal health care system based on the principle of public health insurance. According to law, health insurance is mandatory for all persons with permanent residence in Czechia and persons whose employer has a registered office or permanent residence in Czechia [18]. Funds are collected through insurance premiums paid by employees and their employers or paid by the state. In addition to compulsory health insurance, part of the tax revenue comes from the state budget and direct payments from patients. In Czechia, the principle of solidarity is applied, which means that funds are redistributed between particular insured persons and health insurance companies where they are needed [18, 19].

Health insurance companies manage the founds and provide direct reimbursement of expenses to HSPs. The claim on the disbursement arises on valid contracts between the health insurance company and the HSPs. The aim of concluding contracts between providers and insurance companies is to establish an adequate network of HSPs corresponding to the needs of policyholders and thus ensure available health care in the whole country. Moreover, it seeks to divide competencies between component HSPs and also build on community and social care to achieve high-quality and sustainable health care system.

Individual HSPs offer services depending on the type and form of health care they are appointed with. This paper focuses on outpatient care (or ambulatory care) as health care that does not require an overnight stay in a medical facility. It mainly includes medical consultation, routine physical examinations, procedures, treatment, and others. These services are administered in a variety of different outpatient facilities and are mainly provided by primary healthcare providers (general practitioners, dentists, gynecologists) and specialists. Primary healthcare providers are physicians who provide prevention, diagnosis, and treatment for a wide variety of conditions and illnesses and should be the first point of entry for a patient into the health care system. Specialists are doctors who have advanced training focusing on a specific discipline [18, 19]. Examples of specialists include diabetologists (who treat conditions such as diabetes), cardiologists (studying heart conditions), and psychiatrists (specializing in diagnosis and treatment of mental health issues).

Every patient has the right to receive health services at the appropriate professional level. The availability of healthcare is determined by the travel time to the HSP specified according to the medical specialties or the particular type of services. The maximum travel time is 45 min for outpatient diabetology and 60 min for outpatient cardiology and outpatient psychiatry [20]. According to some previous studies in Czechia [21–23], the travel time is perceived as a highly tolerant limit and basically covers the entire population and territory, assuming transportation by car. Travel time by car or public transport and also distance to HSP are the preferred indicators for most studies because these types of transport are used primary in most (not only developed) countries and allow for the type and condition of the roads [7, 24].

Furthermore, the network of HSPs should ensure their appropriate distribution in space and efficiency of work. For this reason, HSPs occur in areas with a higher population density [25, 26]. This is also proved by our obtained data in Table 1. Most HSPs were concentrated in towns and the smaller the municipality, the more limited the health services availability. The exception is municipalities with 50,000–99,999 inhabitants, which have a smaller proportion of HSPs than do mid-sized municipalities (particularly municipalities with 20,000–49,999 and 10,000–19,999 inhabitants). Furthermore, the outpatient diabetology service providers were more evenly distributed, while outpatient cardiology and psychiatry tended to be concentrated in larger towns.

**TABLE 1:** Health service provider´s structure, Czechia, 2019.

Size of HPS’s municipality	Diabetology		Cardiology		Psychiatry	
	Number	%	Number	%	Number	%
100,000 and more	161	29.1	215	39.2	338	40.6
50,000–99,999	57	10.3	84	15.3	114	13.7
20,000–49,999	112	20.3	112	20.4	155	18.6
10,000–19,999	97	17.5	84	15.3	114	13.7
5,000–9,999	84	15.2	38	6.9	75	9.0
2,000–4,999	35	6.3	13	2.4	20	2.4
less than 2,000	7	1.3	2	0.4	17	2.0
Total	553	100.0	548	100.0	833	100.0

HSP, health service provider.

Data source: GHIC, 2020, own calculations.

## Methods

This type of analysis requires a large amount of detailed information on the use of health services. For this purpose, anonymized individual data from the database of the General Health Insurance Company (GHIC) was used, as it is the only available data source containing this level of detail. Although the input data relate to GHIC insurance-holders only, they represent a sufficiently large sample of the population. GHIC is the largest health insurance company in Czechia, and in 2019 it provided insurance to almost 58% of all health insurance-holders in Czechia [27].

To perform the analysis, knowledge of the patient’s (GHIC insurance-holder’s) age and sex, the code of the municipality (LAU 2) in which the patient was permanently resident, and the municipality code in which the health service was provided. Individuals for whom full data was unavailable were removed from the data set. For all three health services, the resulting data set represented 98.4% of the original data set. The total number of insurance-holders with the full data set was 362,014 for outpatient diabetology, 640,428 for outpatient cardiology, and 324,594 for outpatient psychiatry.

Under the Czech Act on Health Services and Conditions of Their Provision (Act no. 372/2011 Coll.), health service providers (HSPs) can be either legal persons or natural persons authorised to provide health services within the ambit of the law; a patient is a natural person to whom a health service is provided [18]. For the purposes of this article, patients are GHIC insurance-holders who received health care from a selected HSP in 2019.

Therefore, the utilization of health services could be structured according to the size of the patient’s permanent municipality and the size of the HSP’s municipality, only one HSP was selected. Where patients had more than one HSP, the one from which the patient received the most treatment in that year was selected. The distance between the patient’s municipality and the HSP’s municipality was defined as the number of kilometres between the two municipalities (i.e., the “centre” of the municipality). The distance was calculated using Network Analyst in ArcGIS, assuming that the mode of transport used was the car and using expert calculations on average speed for the type of road (see e.g., [28]).

## Results

**FIGURE 1:**
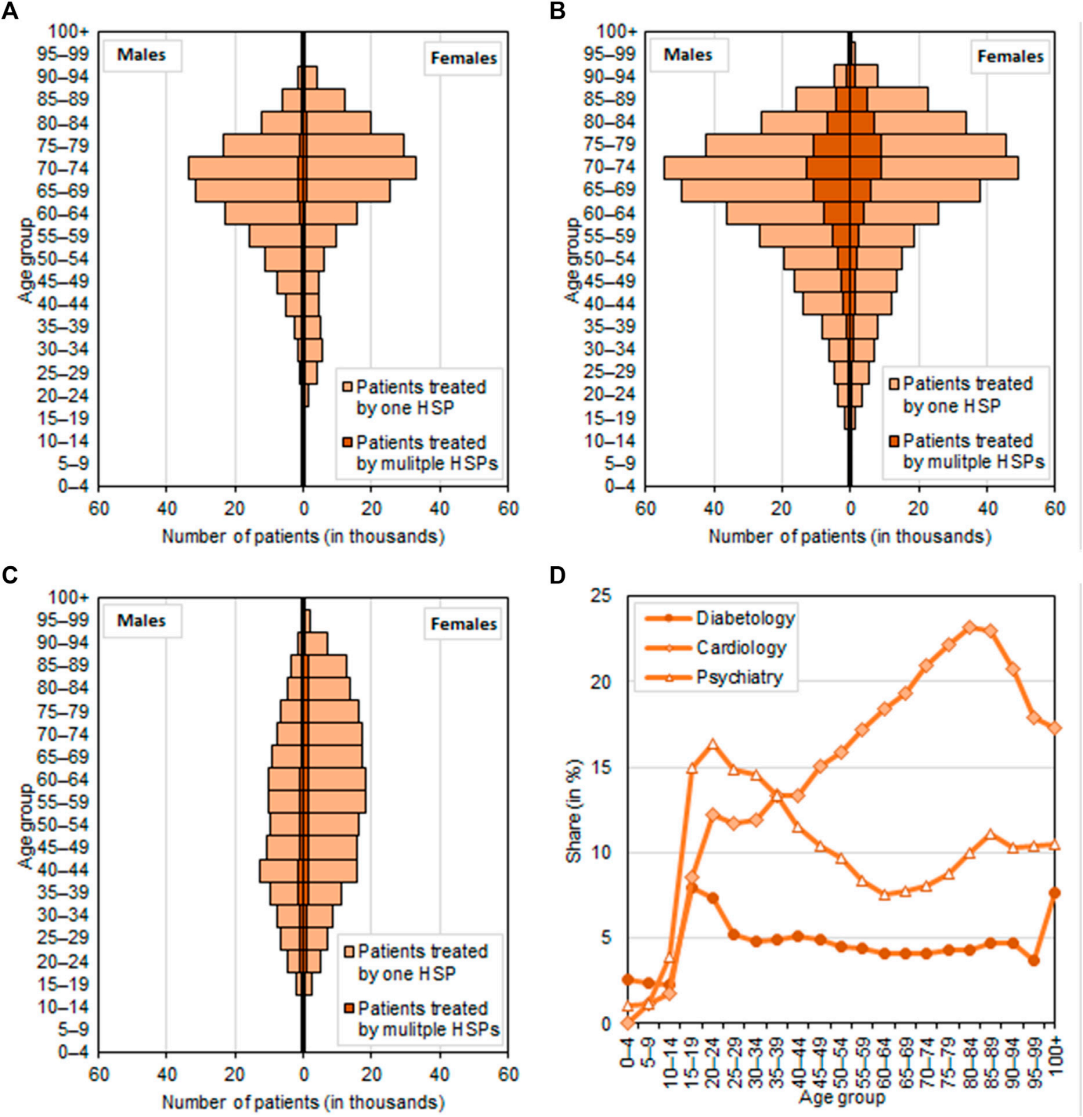
Structure of patient population by sex and age, Czechia, 2019. **(A)**: Diabetology; **(B)**: Cardiology; **(C)**: Psychiatry; **(D)**: Patients treated by multiple HSPs.

Before the results of the analysis of travel for healthcare, the structure of the patient population accesses the relevant health services in 2019 by sex and age is described. The demand for diabetology services (Figure 1A) and cardiology services (Figure 1B) rises with age, with most patients who need these services fall into the 70–74 age category (this applies to both men and women). Patients receiving healthcare in 2019 who were aged 65 and over accounted for 64.4% of diabetology patients and 61.3% of cardiology patients. The proportion of male and female diabetology and cardiology patients was almost equal, but in psychiatry, women represented a larger percentage of patients (Figure 1C) than men did (63.5% of the patients were women). The age structure of psychiatry patients also differed: patients receiving outpatient psychiatry were equally distributed by age. Most male psychiatry patients were aged 40–44, whereas most women were aged 55–59. Therefore, the mean age of patients attending an outpatient psychiatry was 10 years less (56.8 years) than for diabetology and cardiology patients (66.3 years and 65.6 years, respectively) (Table 2).

**TABLE 2:** Basic characteristics of health service utilization, Czechia, 2019.

Indicators	Diabetology			Cardiology			Psychiatry		
	Total	Male	Female	Total	Male	Female	Total	Male	Female
Number of patients	362,014	179,106	182,908	640,428	330,283	310,145	324,594	118,549	206,045
aged 39 and under (%)	6.9	4.1	9.7	7.8	7.4	8.2	20.5	26.7	17.0
aged 40–64 (%)	28.6	35.2	22.2	30.9	34.1	27.4	42.4	44.8	41.1
aged 65 and over (%)	64.4	60.7	68.1	61.3	58.5	64.4	37.0	28.5	42.0
Mean patient age (in years)	66.3	65.6	67.0	65.6	64.6	66.6	56.8	52.5	59.3
Patients receiving healthcare from more than one HSP (in %)	4.4	4.7	4.0	19.2	21.6	16.5	10.3	10.9	9.9
aged 39 and under	5.1	6.5	4.5	12.0	13.0	11.1	14.3	14.5	14.1
aged 40–64	4.4	4.8	3.9	16.5	19.2	12.9	9.5	10.1	9.1
aged 65 and under	4.2	4.6	3.9	21.4	24.1	18.8	9.0	8.9	9.1
Mean number of procedures per patient	6.6	6.9	6.4	4.1	4.4	3.8	6.3	6.7	6.1
aged 39 and under	6.3	7.5	5.8	2.8	2.9	2.6	7.5	7.6	7.5
aged 40–64	6.9	7.1	6.7	3.7	4.0	3.3	6.9	7.4	6.6
aged 65 and over	6.6	6.8	6.4	4.5	4.8	4.2	5.0	4.8	5.0
Patients accessing healthcare at their primary HSP in municipality of residence (in %)	50.8	49.7	51.8	46.5	44.5	48.6	45.0	43.2	46.0
aged 39 and under	40.7	39.7	41.0	41.5	40.5	42.4	41.1	40.0	42.2
aged 40–64	46.7	45.9	48.1	43.4	41.5	45.9	44.0	43.0	44.6
aged 65 and over	53.6	52.6	54.5	48.7	46.7	50.5	48.2	46.7	48.8
Mean distance between patient’s permanent municipality and HSP’s municipality (in km)	10.0	10.7	9.4	15.3	16.5	13.9	16.9	19.6	15.3
aged 39 and under	21.2	24.3	19.8	25.7	26.7	24.8	25.3	26.6	24.2
aged 40–64	12.4	13.2	11.1	17.4	19.1	15.1	16.0	18.8	14.2
aged 65 and over	7.8	8.3	7.4	12.9	13.8	12.0	13.3	14.2	12.9

HSP, health service provider.

Data source: GHIC, 2020, own calculations.

Patients may receive treatment from more than one HSP; nevertheless, most studied patients visited just one HSP. The largest proportion of patients who had multiple HSPs were those receiving cardiology treatment (19.2%). More men than women travelled to receive care provided by multiple HSPs, and this applied to all three types of outpatient service. Diabetology and cardiology patients who most often received treatment with multiple HSPs tended to be older. Travel to access multiple HSPs can also be viewed in terms of the patient’s proportion in the different age categories (Figure 1D). Whereas in diabetology and psychiatry the largest share of patients receiving treatment from multiple HSPs were young patients, in cardiology the opposite was true. Patients aged 65 and over accounted for 21.4% of cardiology patients receiving treatment from multiple HSPs, and the proportion was even higher for 80–84 years olds (Table 2; Figure 1D).

The input data allow us to follow not only the number of patients but also the amount of services provided, by looking at the number of procedures performed per patient per outpatient service, by HSP. The results in Table 2 show that use of service was higher for men than for women for each outpatient service and that there were significant differences in age and service type. The mean number of procedures performed per cardiology patient increases with age of patient, but the opposite is true for psychiatry patients. In diabetology, the differences between the various age categories were smaller than for the other services: among men, the youngest age group had the highest mean number of procedures; among women, it was the age group 40–64.

Healthcare use also differed by patient residency and primary HSP location. As can be seen in Table 3, the larger the municipality, the greater the proportion of medical procedures performed in HSPs in municipalities of that same size; in other words, patients living in smaller municipalities travel to more populous municipalities for their healthcare needs. In municipalities with 100,000 and more inhabitants, the majority of patients (90% and over) accessed health services in municipalities of this size, while this ratio was much lower in municipalities with 5,000–9,999 inhabitants, about half of diabetology patients and a fifth of male cardiology patients. Travel outside of the municipality for healthcare was more common among individuals living in municipalities with fewer than 10,000 inhabitants, who were much more likely to travel to a mid-sized municipality with a population of 20,000–49,999 or 10,000–19,999. Health service utilization was highest in the largest municipalities (100,000 and over inhabitants), followed by municipalities with 20,000–49,999 inhabitants, and lowest in the smallest municipalities. This was the case for both men and women and applied to all three outpatient services.

**TABLE 3:** Structure of health service utilization (number of medical procedures) by size of patient’s permanent municipality and size of health service provider’s municipality (in %), Czechia, 2019.

Categories of municipality in which patient permanently resident	HSP’s municipality categories according to size													
	Diabetology													
	Male							Female						
	100,000 and more	50,000–99,999	20,000–49,999	10,000–19,999	5,000–9,999	2,000–4,999	less than 2,000	100,000 and more	50,000–99,999	20,000–49,999	10,000–19,999	5,000–9,999	2,000–4,999	less than 2,000
100,000 and more	96.2	0.5	0.8	1.2	0.7	0.6	0.1	97.2	0.3	0.6	0.9	0.5	0.5	0.1
50,000–99,999	2.5	93.1	1.4	1.5	0.9	0.5	0.1	2.0	93.9	1.5	1.3	0.7	0.6	0.0
20,000–49,999	3.5	1.9	88.0	2.1	2.0	2.4	0.1	3.1	1.5	89.7	1.5	1.7	2.4	0.0
10,000–19,999	6.6	2.1	7.2	79.4	4.1	0.5	0.2	5.3	1.9	6.8	81.7	3.8	0.4	0.1
5,000–9,999	12.0	8.2	13.6	9.3	52.3	3.7	0.8	10.3	8.1	13.4	9.1	54.9	3.4	0.8
2,000–4,999	15.3	12.3	26.9	20.6	12.0	12.4	0.5	13.2	11.4	27.8	20.9	12.6	13.5	0.6
1,000–1,999	13.8	12.3	25.9	24.4	17.4	5.3	1.0	12.3	12.3	26.6	24.6	17.6	5.5	1.1
500–999	11.7	10.8	23.9	27.2	19.7	5.9	0.7	10.4	10.7	24.5	27.0	20.5	6.2	0.7
less than 500	8.8	10.8	23.4	26.3	23.5	6.4	0.9	7.1	10.4	24.2	26.0	24.5	7.1	0.7
Total	26.8	14.1	22.8	19.0	12.9	4.0	0.4	24.9	14.4	23.6	19.2	13.3	4.1	0.4

	Cardiology													
	Male							Female						
	100,000 and more	50,000–99,999	20,000–49,999	10,000–19,999	5,000–9,999	2,000–4,999	less than 2,000	100,000 and more	50,000–99,999	20,000–49,999	10,000–19,999	5,000–9,999	2,000–4,999	less than 2,000
100,000 and more	95.5	0.6	1.0	1.6	0.7	0.6	0.0	96.6	0.5	0.8	1.1	0.5	0.5	0.0
50,000–99,999	6.1	88.5	3.6	1.2	0.6	0.0	0.0	4.4	91.3	2.9	0.7	0.6	0.0	0.0
20,000–49,999	10.8	4.5	82.6	1.3	0.6	0.1	0.2	8.1	3.5	86.7	1.1	0.5	0.1	0.1
10,000–19,999	15.8	5.5	15.7	57.4	3.7	1.9	0.0	12.6	4.4	14.7	62.5	4.0	1.9	0.0
5,000–9,999	24.4	15.2	22.5	14.4	21.9	1.6	0.0	21.4	14.1	22.5	14.7	25.7	1.6	0.0
2,000–4,999	26.0	16.6	30.4	17.0	6.7	3.3	0.0	22.6	16.1	31.8	18.3	7.3	3.9	0.0
1,000–1,999	24.5	15.0	29.6	21.4	7.4	1.9	0.1	21.7	14.6	30.4	22.5	8.7	1.9	0.1
500–999	23.0	14.1	30.2	21.8	8.8	1.9	0.1	19.9	13.2	31.6	23.1	10.0	2.2	0.0
less than 500	18.5	14.5	30.5	24.1	9.0	3.2	0.1	15.6	13.8	31.7	25.3	9.6	4.1	0.0
Total	36.9	15.6	25.2	15.1	5.6	1.5	0.1	35.4	15.5	25.8	15.6	6.1	1.6	0.0

	Psychiatry													
	Male							Female						
	100,000 and more	50,000–99,999	20,000–49,999	10,000–19,999	5,000–9,999	2,000–4,999	less than 2,000	100,000 and more	50,000–99,999	20,000–49,999	10,000–19,999	5,000–9,999	2,000–4,999	less than 2,000
100,000 and more	89.2	1.4	2.1	2.3	2.7	1.8	0.4	90.6	1.0	1.7	2.1	2.8	1.7	0.1
50,000–99,999	7.0	81.5	5.7	2.3	2.3	0.4	1.0	4.2	87.5	3.9	1.5	2.3	0.2	0.5
20,000–49,999	9.0	5.6	76.6	4.1	3.5	0.5	0.6	6.2	5.0	80.6	3.6	3.9	0.5	0.1
10,000–19,999	13.4	6.4	16.2	56.5	4.8	1.9	0.7	10.4	5.3	15.7	61.3	5.0	2.1	0.2
5,000–9,999	17.7	11.7	20.6	14.8	30.2	4.0	1.0	14.0	11.0	19.0	15.7	35.3	4.4	0.6
2,000–4,999	22.4	14.6	28.6	18.2	8.9	6.5	0.8	19.4	14.9	29.2	18.4	10.3	7.4	0.5
1,000–1,999	19.2	13.5	30.2	19.6	12.7	3.2	1.7	17.5	13.5	28.2	21.0	15.1	3.6	1.1
500–999	17.7	11.6	27.7	22.4	16.4	3.5	0.5	15.1	11.2	26.8	23.8	18.3	4.2	0.6
less than 500	14.4	11.8	26.3	23.0	18.0	5.3	1.3	11.6	12.2	27.3	22.9	19.6	5.1	1.4
Total	32.7	14.5	24.1	15.5	9.6	2.8	0.8	30.3	14.9	24.2	16.3	10.9	3.0	0.5

HSP, health service provider.

Data source: GHIC, 2020, own calculations.

Compared to outpatient cardiology and psychiatry patients, diabetology patients visited an HSP in the same size municipality far more often, and that was true for all municipal categories. Therefore, healthcare utilization was similar in municipalities of the same size. Furthermore, a notably larger proportion of older patients accessing healthcare within their permanent residence municipality than younger patients (see Table 1). In terms of sex differences are concerned, men travelled slightly more than women.

**FIGURE 2:**
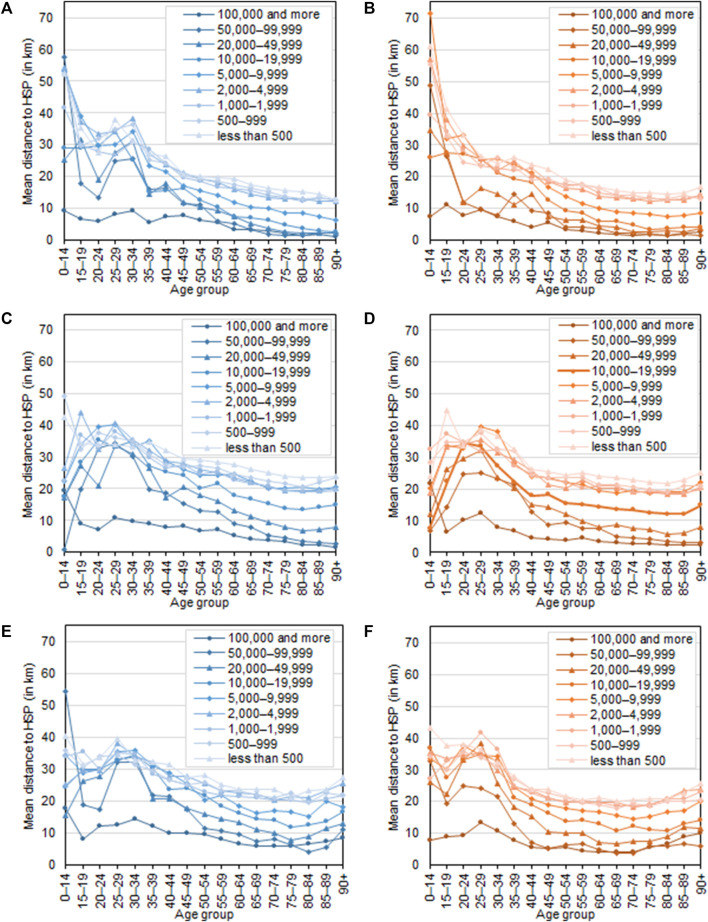
Average distance (in kilometres) between patient’s municipality of permanent residence and municipality in which health service providers accessed in 2019 for each outpatient service, by age, sex and size of patient’s municipality, Czechia, 2019. **(A)**: Diabetology, males; **(B)**: Diabetology, females; **(C)**: Cardiology, males; **(D)**: Cardiology, females; **(E)**: Psychiatry, males; **(F)**: Psychiatry, female.

Geographic information system (GIS) tools were used to calculate the distance between the patient’s municipality and the HSP’s municipality. Figures 2A–F shows the average mean distance patients travelled in 2019 to their primary HSP (where the majority of procedures were performed) by patient’s sex and age and broken down by size of municipality. We can see that patients travel less for healthcare as they get older. Under 34-year-olds travelled the furthest for health care, and this was true for municipalities of all sizes. On average, men travelled more than women, but travel by age group and size of municipality was practically the same for both sex. In terms of size of municipality, the shortest travel distances were found in districts with at least 100,000 inhabitants, while the greatest average distance was found in the smaller municipalities. This trend was evident for all outpatient services.

## Discussion

The structure of the patient sample receiving healthcare by sex and age was largely determined by the structure of the whole population (insurance-holders) and the type of outpatient service. The vast majority of outpatient diabetology and cardiology services were provided to older patients, as the most common diseases affecting elderly people (e.g., diabetes mellitus, hypertension, ischaemic heart disease) come under these services. For example, in 2014 more than a quarter of the Czech population aged 75 and over had diabetes mellitus, dropping to 4% among those aged 45–54 [29]. High blood pressure, the most frequent diagnosis in Czechia, affected almost three in every five people aged 75 and over, but only 23% of those aged 45–54. In contrast, the conditions most frequently treated in outpatient psychiatry (neurosis, stress, somatic, and affective disorders) are not age-specific to the same extent. Our results show that women sought psychiatric care more often than men did, which confirms this trend, with women accounting for 60% of patients over the long term [30].

The analysis also shows that the size of the patient’s municipality of residence has a significant impact on travel for healthcare. Whereas those living in large towns travelled less far and less frequently for healthcare, patients who live in less populous municipalities were far more likely to travel to a more populous municipality for healthcare. Whether the municipality was a local or regional centre also had an effect. HSP location is closely linked to patient healthcare mobility to larger municipalities, and these more populous municipalities have a far wider range of healthcare services per inhabitant. Therefore, the spatial distribution of HSPs explains largely the differences in healthcare care use in terms of the size of municipality (Tables 1, 3). The increasing urban concentration of HSPs, especially specialists, is a long-term phenomenon in Czechia and elsewhere in the world [25, 26, 31]. To reduce inequalities in healthcare access, many policy makers are attempting to reduce imbalances in the spatial distribution of HSPs. One of the many strategies employed to improve the situation in problem areas is to encourage existing doctors to continue practicing in these areas or even to expand their practices. Another one is to motivate novice doctors to set up their practices in areas such as these. However, longitudinal studies have shown that even despite these attempts, most doctors prefer urban locations [32, 33]. On the other hand, we have to recognise that in areas such as those with a low population density, it may that the provision of specialist medical (and other) services is neither effective nor sustainable over the long term [26].

In the present study, the size of the municipality was used to investigate the healthcare use outside the patient’s municipality of residence. A part of the population living in large municipalities, where presumably healthcare provision is sufficient, travels to another municipality for healthcare. The geographic distance to HSP may not be the only factor. Patients can live and use services in a municipality that is not officially their place of permanent residence. Another possible reason could be that patients have their own personal reasons for travelling elsewhere for healthcare, such as choosing somewhere near their place of work or study, recommendations from family or friends, previous experience as a patient, the particular speciality offered by the doctor, a good doctor–patient relationship, the physical appearance of the clinic or the waiting times [34–36]. Although patients are free to choose whichever HSP they want, most patients opt for a compromise between convenience and the choice available. A significant part of the population is willing to travel for healthcare, but only within a certain distance from their place of residence [37]. In our analysis, the mean distance between the place of residence and the location of HSP was a dozen or so kilometres (10.0 km for diabetology, 15.3 km for cardiology and 16.9 km for psychiatry patients; see Table 2). But there were significant differences in the number of kilometres patients travelled depending onsex, age and size of the patient’s municipality of residence. Therefore, the findings support the conclusions about the individual factors in the above mentioned studies.

Longitudinal studies have shown that women generally travel less and for a shorter distance than men for work and other services [38–40]. This study of an example of patients attending diabetology, cardiology, and psychiatry shows the same results. A higher proportion of men than women travelled for healthcare services as well as the average mean distance between patient’s place of residence and the HSP location, was higher for men. Considerable age differences were found. Generally, younger patients were prepared to travel further for healthcare than older patients. On average, the main treatment group travelled the shortest distance from the place of residence to the HSP location. The willingness of older people to commute to a more distant HSP may be limited by their ability and type of transport. A higher proportion of older patients drive less often than younger patients and are more dependent on public transport, which is not always satisfactory, especially in rural areas. Differences in average travel time between car and public transport are substantial. According to Stentzel et al. [3], it is important to consider not only distance, but also transport connections between patients and HSPs to secure adequate access to healthcare. That type of analysis could also be beneficial to Czechia.

Given the ageing Czech population and the long-term growth in the incidence of diabetes mellitus and circulatory diseases, even among younger generations, it is possible that the number of patients and the demand for diabetology and cardiology services will continue to increase. This could in turn affect the level of healthcare utilization in certain areas and possibly lead to increased travel for these health services. Recent international studies have shown that, in high-income countries, although the proportion of people being diagnosed, treated, and receiving effective treatment has been rising over the long term, there is still room for improvement [41]. Approximately a fifth to a third of those with hypertension do not know about their condition. Even in countries with the best healthcare outcomes, at least a fifth of people receive no treatment, and effective treatment was demonstrated in less than 60% of cases [41, 42].

### Strengths and Limitations

This study has several strengths and limitations. First of all, it is worth noting that although many studies have dealt with the use of healthcare services in other countries, in Czechia this issue—especially healthcare accessibility—has received insufficient attention, despite being a serious and pressing issue. The present study was conducted using a large amount of anonymized individual data containing a detailed level of information, making it possible to conduct an extensive and thorough analysis of healthcare accessibility. As noted at the beginning, the data were collected by GHIC, a health insurance company covering at least three-fifths of health insurance-holders in Czechia. Health insurance is obligatory in Czechia and is used to cover the costs of providing health care to the insurance-holder. Anyone who is a permanent resident of Czechia is legally obliged to have health insurance. Therefore, this study is based on a large sample of the whole population. The limitation of this is that older insurance-holders are more likely to be insured with GHIC than with other insurance companies, which could introduce an element of bias. On the other hand, the sample captures a larger proportion of elder patients with higher risk.

Another limitation concerns the geographic accessibility of health services and the use of the patient’s permanent residence to study this: in reality, the patient may be ordinarily resident elsewhere. In practice, patients can live and receive healthcare in the same municipality, but because they are officially registered in another municipality for administrative purposes, they appear to travel to a different municipality for healthcare. Unfortunately, as there is no available register or database containing information on ordinary residence (and this applies to our data source as well), the research has no choice but to rely on the permanent residency data.

### Conclusion

The analysis shows important differences in the utilization of selected outpatient health services by age, sex and size of municipality. Although the sex and age structure of the patients differed substantially for the various outpatient services, the trend in healthcare utilization was similar. Men were slightly more likely to travel for healthcare than women, and the average distance from the place of residence to the HSP location decreased with the age of the patient. Diabetology, cardiology, and psychiatry patients permanently resident in a larger municipality were more likely to make use of outpatient health services in a similarly sized municipality, whereas patients living in smaller municipalities were more likely to travel to access health services. Additionally, the mean number of kilometres that patients travelled from their place of residence to the HSP location increased with smaller municipalities. This was largely due to the unequal distribution of the HSPs studied, which tended to be concentrated in larger municipalities and local and regional centres. Simultaneously, other factors, such as individual factors, are assumed to play a role.

## References

[1] Gulliford M Figueroa-Munoz J Morgan M Hughes D Gibson B Beech R What Does 'access to Health Care' Mean. J Health Serv Res Pol (2002) 7(3):186–8. 10.1258/135581902760082517 12171751

[2] Fitzpatrick AL Powe NR Cooper LS Ives DG Robbins JA. Barriers to Health Care Access Among the Elderly and Who Perceives Them. Am J Public Health (2004) 94(10):1788–94. 10.2105/AJPH.94.10.1788 15451751 PMC1448535

[3] Stentzel U Piegsa J Fredrich D Hoffmann W van den Berg N. Accessibility of General Practitioners and Selected Specialist Physicians by Car and by Public Transport in a Rural Region of Germany. BMC Health Serv Res (2016) 16(587). 10.1186/s12913-016-1839-y PMC5070365 27756338

[4] Aday LA Andersen R. A Framework for the Study of Access to Medical Care. Health Serv Res (1974) 9(3):208–20. 4436074 PMC1071804

[5] Steinwachs DM Hughes RG. Health Services Research: Scope and Significance. In: Hughes RG, editor. Patient Safety and Quality: An Evidence-Based Handbook for Nurses. Rockville (MD): Agency for Healthcare Research and Quality (2008). p. 163–77.

[6] Bourke L Humphreys JS Wakerman J Taylor J. Understanding Rural and Remote Health: A Framework for Analysis in Australia. Health & Place (2012) 18(3):496–503. 10.1016/j.healthplace.2012.02.009 22418016

[7] Yerramilli S Fonseca DG. Assessing Geographical Inaccessibility to Health Care: Using GIS Network Based Methods. Public Health Res (2014) 4(5):145–59. 10.5923/j.phr.20140405.01

[8] Andersen R Newman JF. Societal and Individual Determinants of Medical Care Utilization in the United States. Milbank Q (2005) 83(4):Online–only. 10.1111/j.1468-0009.2005.00428.x 4198894

[9] David JL Kaplan HB. Gender, Social Roles and Health Care Utilization. Appl Behav Sci Rev (1995) 3(1):39–64. 10.1016/S1068-8595(95)80012-3

[10] Parkash J Younis MZ Ward W. Healthcare for the Ageing Populations of Countries of Middle East and North Africa. Ageing Int (2015) 40(1):3–12. 10.1007/s12126-012-9150-7

[11] Seshamani M Gray A. Time to Death and Health Expenditure: an Improved Model for the Impact of Demographic Change on Health Care Costs. Age and Ageing (2004) 33(6):556–61. 10.1093/ageing/afh187 15308460

[12] Šídlo L Šprocha B Ďurček P. A Retrospective and Prospective View of Current and Future Population Ageing in the European Union 28 Countries. Moravian Geographical Rep (2020) 28(3):187–207. 10.2478/mgr-2020-0014

[13] Šídlo L Šprocha B Klapková M. Regional Differences in Population Aging in Europe Viewed through Prospective Indicators. Erdkunde (2019) 73(3):225–40. 10.3112/erdkunde.2019.03.06

[14] Arber S. Comparing Inequalities in Women's and Men's Health: Britain in the 1990s. Soc Sci Med (1997) 44(6):773–87. 10.1016/S0277-9536(96)00185-2 9080561

[15] Mustard CA Kaufert P Kozyrskyj A Mayer T. Sex Differences in the Use of Health Care Services. N Engl J Med (1998) 338(23):1678–83. 10.1056/NEJM199806043382307 9614260

[16] Bertakis KD Azari R Helms LJ Callahan EJ Robbins JA. Gender Differences in the Utilization of Health Care Services. J Fam Pract (2000) 49(2):147–52. 10718692

[17] Badji S Badland H Rachele JN Petrie D. Public Transport Availability and Healthcare Use for Australian Adults Aged 18-60 Years, with and without Disabilities. J Transport Health (2021) 20:101001. 10.1016/j.jth.2020.101001

[18] Česko. Zákon č. 372/2011 Sb. Zákon O Zdravotních Službách a Podmínkách Jejich Poskytování (Zákon O Zdravotních Službách). Aktuální Znění 01.10.2020-31.12.2020 (Verze 24) [Act No. 372/2011 Coll., on Health Services and Conditions of Their Provision (Act on Health Services). Current Version 01.10.2020-31.12.2020 (Version 24)]. AION CS 2010-2020 (2011). Available from: https://www.zakonyprolidi.cz/cs/2011-372 (Acceessed October 30, 2020).

[19] Janečková H Hnilicová H. Úvod Do Veřejného Zdravotnictví. Portál: Praque (2009). p. 294.

[20] Česko. Nařízení Vlády Č. 307/2012 Sb. Nařízení Vlády O Místní a Časové Dostupnosti Zdravotních Služeb. Aktuální Znění 01.01.2013 (Verze 1) [Government Regulation No. 307/2012 Coll. Government Regulations on Local and Time Availability of Health Services. Current Version 01.01.2013 (Version 1)]. AION CS 2010-2020 (2011). Available from: https://www.zakonyprolidi.cz/cs/2012-307 (Acceessed November 20, 2021).

[21] Štych P Šaffová M Šídlo L. Místní Dostupnost Zdravotních Ambulantních Služeb V Česku. Ambulantní Diabetologie. Prague: Nakladatelství P3K (2020). p. 10. Available from: https://drive.google.com/file/d/1iXPOzJ5ZbTVlSYjDhgAl0ltpaG-Rj7V2G/view (Acceessed November 20, 2021).

[22] Štych P Šaffová M Šídlo L. Místní Dostupnost Zdravotních Ambulantních Služeb V Česku. Ambulantní Kardiologie. Prague: Nakladatelství P3K (2020). p. 10. Available from: https://drive.google.com/file/d/1GVahk6ULLQwPVoK-8LBZhSjKWThtEvMI/view (Acceessed November 20, 2021).

[23] Štych P Šaffová M Šídlo L. Místní Dostupnost Zdravotních Ambulantních Služeb V Česku. Ambulantní Psychiatrie. Prague: Nakladatelství P3K (2020). p. 10. Available from: https://drive.google.com/file/d/1oD4EGv4gsJj8cXHrgTORuDk9o8iuibDn/view?usp=sharing (Acceessed November 20, 2021).

[24] Kara F Egresi I. Accessibility of Health Care Institutions: A Case Study by Using GIS. Int J Scientific Knowledge (2013) 3(4):16–27.

[25] Šídlo L Bělobrádek J Maláková K. General Medical Practitioners in Czechia: Development Trends and Regional Differences. Geografie (2021) 126(2):169–94. 10.37040/geografie2021126020169

[26] Ono T Schoenstein M Buchan J Geographic Imbalances in Doctor Supply and Policy Responses. OECD Health Working Papers, 69. Paris: OECD Publishing (2014). p. 65. 10.1787/5jz5sq5ls1wl-en

[27] General Health Insurance Company (GHIC). Data for the Project No. TL01000382. GHIC (2020).

[28] Hudeček T. Dostupnost V Česku V Období 1991-2001: Vztah K Dojížďce Do Zaměstnání a Do Škol. Prague: Česká geografická společnost (2010). p. 141

[29] Eurostat. Database. Persons Reporting a Chronic Disease, by Disease, Sex, Age and Educational Attainment Level (2020). Available from: https://ec.europa.eu/eurostat/data/database (Accessed October 31, 2020).

[30] Institute of Health Information and Statistics of the Czech Republic (IHIS). Psychiatrická Péče 2018. Prague: IHIS (2019). p. 122.

[31] Health Policy Institute. Rural and Urban Health (2020). Available from: https://hpi.georgetown.edu/rural/ (Accessed October 31, 2020).

[32] Natanzon I Szecsenyi J Ose D Joos S. Future Potential Country Doctor: the Perspectives of German GPs. Rural Remote Health (2010) 10(2):1347. 10.22605/rrh1347 20455635

[33] Weinhold I Gurtner S. Understanding Shortages of Sufficient Health Care in Rural Areas. Health Policy (2014) 118(2):201–14. 10.1016/j.healthpol.2014.07.018 25176511

[34] Bornstein BH Marcus D Cassidy W. Choosing a Doctor: an Exploratory Study of Factors Influencing Patients' Choice of a Primary Care Doctor. J Eval Clin Pract (2000) 6(3):255–62. 10.1046/j.1365-2753.2000.00256.x 11083036

[35] Liu N Finkelstein SR Kruk ME Rosenthal D. When Waiting to See a Doctor Is Less Irritating: Understanding Patient Preferences and Choice Behavior in Appointment Scheduling. Manage Sci (2018) 64(5):1975–96. 10.1287/mnsc.2016.2704

[36] Mercado F Mercado M Myers N Hewit M Haller NA. Patient Preferences in Choosing a Primary Care Physician. J Prim Care Community Health (2012) 3(2):125–31. 10.1177/2150131911421802 23803456

[37] McGrail MR Humphreys JS. Measuring Spatial Accessibility to Primary Health Care Services: Utilising Dynamic Catchment Sizes. Appl Geogr (2014) 54:182–8. 10.1016/j.apgeog.2014.08.005

[38] White MJ. A Model of Residential Location Choice and Commuting by Men and Women Workers. J Reg Sci (1977) 17(1):41–52. 10.1111/j.1467-9787.1977.tb00471.x 12337077

[39] Fanning Madden J. Why Women Work Closer to Home. Urban Stud (1981) 18(2):181–94. 10.1080/00420988120080341

[40] McLafferty S Preston V. Gender, Race, and Commuting Among Service Sector Workers∗. The Prof Geographer (1991) 43(1):1–15. 10.1111/j.0033-0124.1991.00001.x

[41] Zhou B Danaei G Stevens GA Bixby H Taddei C Carrillo-Larco RM Long-term and Recent Trends in Hypertension Awareness, Treatment, and Control in 12 High-Income Countries: an Analysis of 123 Nationally Representative Surveys. The Lancet (2019) 394(10199):639–51. 10.1016/S0140-6736(19)31145-6 PMC6717084 31327564

[42] Joffres M Falaschetti E Gillespie C Robitaille C Loustalot F Poulter N Hypertension Prevalence, Awareness, Treatment and Control in National Surveys from England, the USA and Canada, and Correlation with Stroke and Ischaemic Heart Disease Mortality: a Cross-Sectional Study. BMJ Open (2013) 3:3. 10.1136/bmjopen-2013-003423 PMC3758966 23996822

